# Relationship between carotid intima-media thickness and coronary angiographic findings: a prospective study

**DOI:** 10.1186/1476-7120-7-59

**Published:** 2009-12-31

**Authors:** Ugur Coskun, Ahmet Yildiz, Ozlem B Esen, Murat Baskurt, Mehmet A Cakar, Kadriye O Kilickesmez, Lutfu A Orhan, Seyma Yildiz

**Affiliations:** 1 Istanbul University, Institute of Cardiology, Haseki, Istanbul, Turkey; 2 Sakarya Training and Research Hospital Department of Cardiology, Sakarya, Turkey; 3 Siyami-Ersek Training and Research Hospital, Department of Cardiology, Istanbul, Turkey; 4 Bagcilar Training and Research Hospital, Department of Radiology, Istanbul, Turkey

## Abstract

**Background:**

Since cardiovascular diseases are associated with high mortality and generally undiagnosed before the onset of clinical findings, there is a need for a reliable tool for early diagnosis. Carotid intima-media thickness (CIMT) is a non-invasive marker of coronary artery disease (CAD) and is widely used in practice as an inexpensive, reliable, and reproducible method. In the current study, we aimed to investigate prospectively the relationship of CIMT with the presence and extent of significant coronary artery narrowing in patients evaluated by coronary angiography for stable angina pectoris.

**Methods:**

One hundred consecutive patients with stable angina pectoris and documented ischemia on a stress test were included in the study. The patients were divided into two groups according to the result of the coronary angiography: group 1 (39 patients) without a noncritical coronary lesion, and group 2 (61 patients) having at least one lesion more than 50% within the main branches of the coronary arteries. All of the patients underwent carotid Doppler ultrasound examination for measurement of the CIMT by a radiologist blinded to the angiographic data.

**Results:**

The mean CIMT was 0.78 ± 0.21 mm in Group 1, while it was 1.48 ± 0.28 mm in Group 2 (p = 0.001). The mean CIMT in patients with single vessel disease, multi-vessel disease, and left main coronary artery disease were significantly higher compared to Group 1 (1.2 ± 0.34 mm, p = 0.02; 1.6 ± 0.32 mm, p = 0.001; and 1.8 ± 0.31 mm, p = 0.0001, respectively). Logistic regression analysis identified CIMT (OR 4.3, p < 0.001) and hypertension (OR 2.4, p = 0.04) as the most important factors for predicting CAD.

**Conclusions:**

The findings of this study show that increase in CIMT is associated with the presence and extent of CAD. In conclusion, we demonstrated the usefulness of carotid intima-media thickness in predicting coronary artery disease but large-scale studies are required to define its role in clinical practice.

## Background

Cardiovascular-related deaths are the leading cause of death both in Turkey and worldwide [1-3]. At the time of onset of clinical findings, an advanced stage of involvement is often present in atherosclerotic disease. Thus, limited benefit can be obtained from various interventions beyond that point. Many significant changes occur in the arterial wall, including endothelial dysfunction and an increase in intima-media thickness, before the onset of clinical findings, and these changes can be useful in the early diagnosis of atherosclerosis [4,5].

After the first report of intima-media thickness measurement by Pignoli et al. [6], rapid progress has been noted in ultrasonographic imaging methods and carotid intima-media thickness (CIMT) has begun to be used as an inexpensive, reliable, and reproducible method in the diagnosis of atherosclerosis [7,8].

In the current study, we aimed to investigate the relationship between CIMT and coronary artery disease (CAD) in patients evaluated by coronary angiography for suspected CAD.

## Methods

One hundred consecutive patients referred to our cardiology outpatient clinic with stable angina pectoris and documented ischemia on a stress test were included in the study. Patients with previous myocardial infarction, coronary-artery bypass graft operation and stroke were excluded. Informed consent was obtained from all of the patients. The patients were then scheduled for coronary angiography and carotid doppler ultrasound examination. All of the patients underwent carotid doppler ultrasound examination for measurement of the CIMT by a single radiologist blinded to the angiographic result.

All of the carotid scans were done by an ultrasonography device (General Electric Logic 9; USA) equipped with a 10 mHz linear probe. The CIMT was measured 1 cm distal to the bulbus over a length of 1 cm of both carotid arteries. Manual measurements in five different locations on the far-wall were taken and the maximum value was taken for each carotid artery. The final CIMT was evaluated as the average of right and left carotid arteries [9].

Coronary angiography was performed by the standard Judkins method using a General Electric System 2000, and the results were evaluated by one experienced interventional cardiologist. Luminal narrowing at least one lesion > 50% in the within the main branches of coronary artery was considered as significant CAD.

### Statistical analysis

Statistical analysis was conducted using the SPSS 11.0 program (SPSS Inc., IL, Chicago, USA). Quantitative variables were expressed as mean ± standard deviation and qualitative variables as percent. Student t-test or Mann-Whitney U-test were used for between-group comparisons of continuous variables (according to distribution characteristics) while the chi-square test was used for between-group comparisons of categorical variables. Correlation coefficients >0.05 were considered to be significant. Bivariate logistic regression models were used to identify independent risk factors for CAD. Logistic regression analysis (with CAD as the outcome variable) was performed to determine whether CIMT was an independent predictor of CAD.

## Results

**Figure 1:**
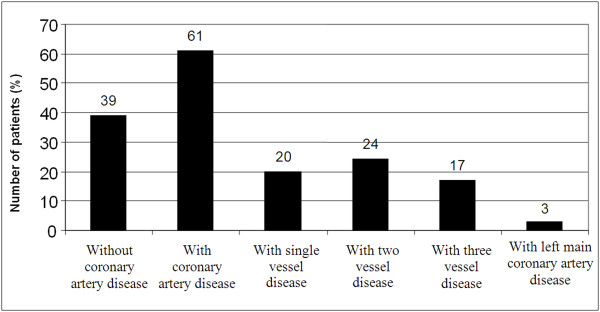
**Coronary angiographic features of the patients included in the study**.

**Figure 2:**
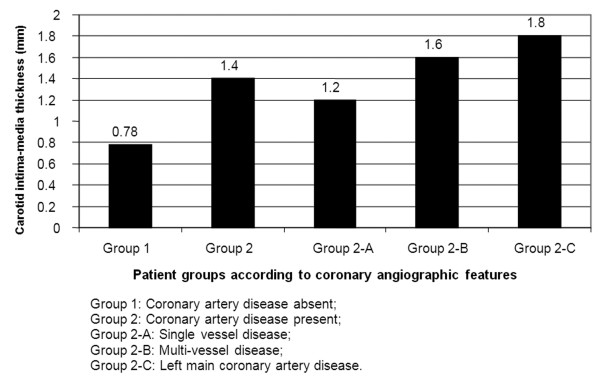
**Comparison of carotid intima-media thickness with coronary angiographic findings**.

While no significant narrowing was noted in coronary arteries in 39 of 100 (39%) patients evaluated by coronary angiography, significant narrowing was noted in 61 patients (61%). The demographic characteristics, the extent of CAD, and the distribution of the mean CIMT values in both groups are presented in Table 1 and in Figures 1 and 2. There were significantly more hypertensive, diabetic, and hyperlipidemic patients in Group 2 (p < 0.05). There were no significant differences between the two groups in terms of age, gender, and smoking habits.

**Table 1:** Demographic characteristics of the patients.

Characteristics	Group 1 (n = 39)	Group 2 (n = 61)	P
**Age (mean ± SD)**	57 ± 9	61 ± 10	NS
**Female gender n (%)**	16 (41)	22 (36)	NS
**BMI in kg/m^2 ^(mean ± SD)**	26	36	NS
**Smoking n (%)**	21 (54)	37 (60)	NS
**Hypertension n (%)**	14 (36)	38 (62)	0.01
**Diabetes n (%)**	4 (10)	19 (31)	0.01
**Hyperlipidemia n (%)**	7 (18)	30 (49)	0.01

**BMI**: body mass index; **NS**: not significant; **CAD**: coronary artery disease

The mean CIMT was 0.78 ± 0.21 mm in Group 1, and 1.48 ± 0.28 mm in Group 2 (p = 0.001). The mean CIMT was statistically significantly higher in the groups with single vessel disease, multi-vessel disease, and left main coronary artery disease compared to Group 1 (1.2 ± 0.34 mm, p = 0.02; 1.6 ± 0.32 mm, p = 0.001; and 1.8 ± 0.31 mm, p = 0.0001, respectively; Figure 2).

A regression model was created using variables that were significantly associated with CAD on univariate analysis, CIMT (>1.0 mm), hypertension, diabetes, and hyperlipidemia. Backward stepwise analysis on this model identified CIMT (OR 4.3, p < 0.001) and hypertension (OR 2.4, p = 0.04) as the most important factors for predicting CAD (Table 2).

**Table 2:** Logistic regression model for statistically significant risk factors predicting coronary artery disease

Variables	P value	Odds Ratio
**CIMT (>1.0 mm)**	0,0001	4,3
**Hypertension**	0,040	2,4
**Diabetes**	0,647	0,730
**Hyperlipidemia**	0,593	1,416

**CIMT**: carotid intima-media thickness

## Discussion

Interventional and non-interventional methods to detect atherosclerosis are widely used in clinical practice. CIMT measurement has been recommended by the American Heart Association as the most useful method to identify atherosclerosis [10]. CIMT can be measured by B-mode ultrasonography.

There are several prospective epidemiologic studies including the Atherosclerosis Risk in Communities (ARIC) Study [11] and the Cardiovascular Health Study (CHS) [12], which have supported a direct correlation of CIMT with myocardial infarction and stroke risk in patients without cardiovascular disease history. The ARIC Study was conducted in 15,792 individuals between 5 and 65 years of age in 4 different regions of the USA between 1987 and 1989. The baseline CIMT was measured and measurements were repeated at 4-7 year intervals by carotid B mode ultrasonography in this study. An increase in CIMT was correlated with an increased risk for CAD. The CHS was initiated in 1988, and the relationship of CIMT with risk of myocardial infarction and stroke was investigated in 4,476 subjects ≤65 years of age. At the end of approximately 6 years of follow-up, CIMT measurements were correlated with cardiovascular events.

Paroi artérielle et Risque Cardiovasculaire in Asia Africa/Middle East and Latin America (PARC-AALA) is another important large-scale study, in which 79 centers from countries in Asia, Africa, the Middle East, and Latin America participated, and the distribution of CIMT according to different ethnic groups and its association with the Framingham cardiovascular score was investigated [13]. Multi-linear regression analysis revealed that an increased Framingham cardiovascular score was associated with CIMT, and carotid plaque independent of geographic differences.

Cahn et al. [14] prospectively followed-up 152 patients with coronary artery disease for 6-11 months by carotid artery ultrasonography and noted 22 vascular events (myocardial infarction, transient ischemic attack, stroke, and coronary angioplasty) within this time period. They concluded that carotid atherosclerosis measured by this non-interventional method has prognostic significance in coronary artery patients.

In the Rotterdam study, Bots et al. [15] followed 7,983 patients >55 years of age for a mean period of 4.6 years, and reported 194 incident myocardial infarctions within this period. CIMT was significantly higher in the myocardial infarction group compared to the other group.

In a study from our country, Demircan et al. [16] found that the CIMT of patients with acute coronary syndrome were significantly increased compared to patients with stable angina pectoris. It has been reported in another study that a maximal CIMT value of 0.956 mm had 85.7% sensitivity and 85.1% specificity to predict angiographic CAD [9].

Our study group consisted of patients admitted to the cardiology outpatient clinic with symptoms of stable angina pectoris. The present study showed CIMT was higher in patients with significant CAD than in patients with noncritical coronary lesions. Regression analysis revealed that thickening of the mean intima-media complex more than 1.0 was predictive of significant CAD our patients. There was incremental significant increase in CIMT with the number coronary vessel involved.

In accordance with the literature, we found that CIMT was significantly higher in the presence of CAD. Furthermore, CIMT was increased as the number of involved vessels increased and the highest CIMT values were noted in patients with left main coronary involvement.

## Limitations

Our study group was small due to involving a single center and symptomatic patients. Since the study was cross-sectional in design, the clinical endpoints were not followed.

## Conclusions

The findings of this study show that increase in CIMT is associated with the presence and extent of CAD. Carotid Doppler ultrasonography can be utilized as a valuable screening tool due to its several advantages, including ease of application, reproducibility, low cost and strong correlation with atherosclerosis. In conclusion, we demonstrated the usefulness of carotid intima-media thickness in predicting coronary artery disease but large-scale studies are required to define its role in clinical practice.

## Competing interests

The authors declare that they have no competing interests.

## Authors' contributions

UC: Participated in performance of coronary angiograms, data analysis and drafting of the manuscript.

AY: Participated in performance and interpretation of coronary angiograms and drafting of the manuscript.

OBE: Participated in data collection, drafting and final revision of the manuscript.

MB: Participated in data collection and coordination of the study.

MAC: Performed the statistical analysis

KOK: Participated in data collection and coordination of the study.

LAO: Participated in data collection and coordination of the study.

SY: Performed the carotid Doppler examinations

All authors read and approved the final manuscript.

## References

[B1] Murray CJ Lopez AD Mortality by cause for eight regions of the world: Global Burden of Disease Study Lancet 1997 349 1269 1276 10.1016/S0140-6736(96)07493-4 9142060

[B2] Gaziano JM Braunwald E, Zipes DP, Libby P Global burden of cardiovascular disease Heart disease: A textbook of cardiovascular medicine 2001 6 Philadelphia: WB Saunders Company 1 17

[B3] Onat A Sansoy V Soydan Tokgozoglu L Adalet K TEKHARF; On iki yillik izleme deneyimine gore Turk eriskinlerinde kalp sagligi 2003 Istanbul: ARGOS Iletisim

[B4] Kanters SD Algra A van Leeuwen MS Banga JD Reproducibility of in vivo carotid intima-media thickness measurements: a review Stroke 1997 28 665 671 9056629 10.1161/01.str.28.3.665

[B5] Kuller L Borhani N Furberg C Gardin J Manolio T O'Leary D Psaty B Robbins J Prevalence of subclinical atherosclerosis and cardiovascular disease and association with risk factors in the Cardiovascular Health Study Am J Epidemiol 1994 139 1164 1179 8209875 10.1093/oxfordjournals.aje.a116963

[B6] Pignoli P Tremoli E Poli A Oreste P Paoletti R Intimal plus medial thickness of the arterial wall: a direct measurement with ultrasound imaging Circulation 1986 74 1399 1406 3536154 10.1161/01.cir.74.6.1399

[B7] O'Leary DH Polak JF Intima-media thickness: a tool for atherosclerosis imaging and event prediction Am J Cardiol 2002 90 18L 21L 10.1016/S0002-9149(02)02957-0 12459422

[B8] Greenland P Abrams J Aurigemma GP Bond MG Clark LT Criqui MH Crouse JR Friedman L Fuster V Herrington DM Kuller LH Ridker PM Roberts WC Stanford W Stone N Swan HJ Taubert KA Wexler L Prevention Conference V: Beyond secondary prevention: identifying the high-risk patient for primary prevention: noninvasive tests of atherosclerotic burden: Writing Group III Circulation 2000 101 E16 E22 10618318 10.1161/01.cir.101.1.e16

[B9] Altekin ER Demir I Basarici I Yilmaz H The relationship between carotid intima-media thickness and the presence and extent of angiographic coronary artery disease Turk Kardiyol Dern Ars 2007 35 90 96

[B10] Smith SC Jr Greenland P Grundy SM AHA Conference Proceedings. Prevention conference V: Beyond secondary prevention: Identifying the high-risk patient for primary prevention: executive summary. American Heart Association Circulation 2000 101 111 116 10618313 10.1161/01.cir.101.1.111

[B11] Chambless LE Heiss G Folsom AR Rosamond W Szklo M Sharrett AR Clegg LX Association of coronary heart disease incidence with carotid arterial wall thickness and major risk factors: the Atherosclerosis Risk in Communities (ARIC) Study, 1987-1993 Am J Epidemiol 1997 146 483 494 9290509 10.1093/oxfordjournals.aje.a009302

[B12] O'Leary DH Polak JF Kronmal RA Manolio TA Burke GL Wolfson SK Jr Carotid-artery intima and media thickness as a risk factor for myocardial infarction and stroke in older adults. Cardiovascular Health Study Collaborative Research Group N Engl J Med 1999 340 14 22 10.1056/NEJM199901073400103 9878640

[B13] Touboul PJ Hernandez-Hernández R Kucukoglu S Woo KS Vicaut E Labreuche J Migom C Silva H Vinueza R PARC-AALA Investigators Carotid artery intima media thickness, plaque and Framingham cardiovascular score in Asia, Africa/Middle East and Latin America: the PARC-AALA study Int J Cardiovasc Imaging 2007 23 557 567 10.1007/s10554-006-9197-1 17186134

[B14] Chan SY Mancini GB Kuramoto L Schulzer M Frohlich J Ignaszewski A The prognostic importance of endothelial dysfunction and carotid atheroma burden in patients with coronary artery disease J Am Coll Cardiol 2003 42 1037 1043 10.1016/S0735-1097(03)00927-6 13678927

[B15] Meer IM Van der Bots ML Hofman A del Sol AI Kuip DA van der Witteman JC Predictive value of noninvasive measures of atherosclerosis for incident myocardial infarction: the Rotterdam Study Circulation 2004 109 1089 1094 10.1161/01.CIR.0000120708.59903.1B 14993130

[B16] Demircan S Tekin A Tekin G Topcu S Yigit F Erol T Katircibasi T Sezgin AT Baltali M Ozin B Müderrisoglu H Comparison of carotid intima-media thickness in patients with stable angina pectoris versus patients with acute coronary syndrome Am J Cardiol 2005 96 643 644 10.1016/j.amjcard.2005.04.035 16125486

